# Ultra-Low Specific On-resistance Lateral Double-Diffused Metal-Oxide-Semiconductor Transistor with Enhanced Dual-Gate and Partial P-buried Layer

**DOI:** 10.1186/s11671-019-2866-5

**Published:** 2019-01-28

**Authors:** Zhuo Wang, Zhangyi’an Yuan, Xin Zhou, Ming Qiao, Zhaoji Li, Bo Zhang

**Affiliations:** State Key Laboratory of Electronic Thin Films and Integrated Devices, University of Electronic Science and Technology of China, Chengdu, 610054 Sichuan China

**Keywords:** Enhanced dual-gate, Lateral double-diffused metal-oxide-semiconductor transistor (LDMOS), Partial buried layer, Specific on-resistance

## Abstract

An ultra-low specific on-resistance (*R*_on,sp_) lateral double-diffused metal-oxide-semiconductor transistor (LDMOS) with enhanced dual-gate and partial P-buried layer is proposed and investigated in this paper. On-resistance analytical model for the proposed LDMOS is built to provide an in-depth insight into the relationship between the drift region resistance and the channel region resistance. N-buried layer is introduced under P-well to provide a low-resistance conduction path and reduce the resistance of the channel region significantly. Enhanced dual-gate structure is formed by N-buried layer while avoiding the vertical punch-through breakdown in off-state. Partial P-buried layer with optimized length is adopted under the N-drift region to extend vertical depletion region and relax the electric field peak in off-state, which enhances breakdown voltage (BV) with low drift region resistance. For the LDMOS with enhanced dual-gate and partial P-buried layer, the result shows that *R*_on,sp_ is 8.5 mΩ·mm^2^ while BV is 43 V.

## Background

With the increase of demand for more complex and faster logic function in analog power IC, it is significant to improve the performance of the lateral double-diffused metal-oxide-semiconductor transistor (LDMOS), specially minimizing specific on-resistance (*R*_on,sp_) and maximizing off-state breakdown voltage (BV) [1–9]. Most developed technologies focus on the drift region optimizing to improve the trade-off of *R*_on,sp_ vs. BV for LDMOS devices [10–20]. In our previous work, the LDMOS with ultra-shallow trench isolation (USTI) was proposed [21]. The depth and corner angel of USTI were optimized to achieve best-in-class performance. However, for the low voltage LDMOS, the drift region is losing domination in *R*_on,sp_ and the contribution of the channel region cannot be ignored.

## Method

In this work, a novel ultra-low specific on-resistance LDMOS with enhanced dual-gate and partial P-buried layer is investigated. The physical models IMPACT.I, BGN, CONMOB, FLDMOB, SRH, and SRFMOB are used in numerical simulation. On-resistance analytical model is proposed to provide an in-depth insight into the relationship between the drift region resistance and the channel region resistance. Based on the model, N-buried layer and partial P-buried layer are optimized to achieve low *R*_on,sp_ and high BV.

## Results and Discussion

**Fig. 1:**
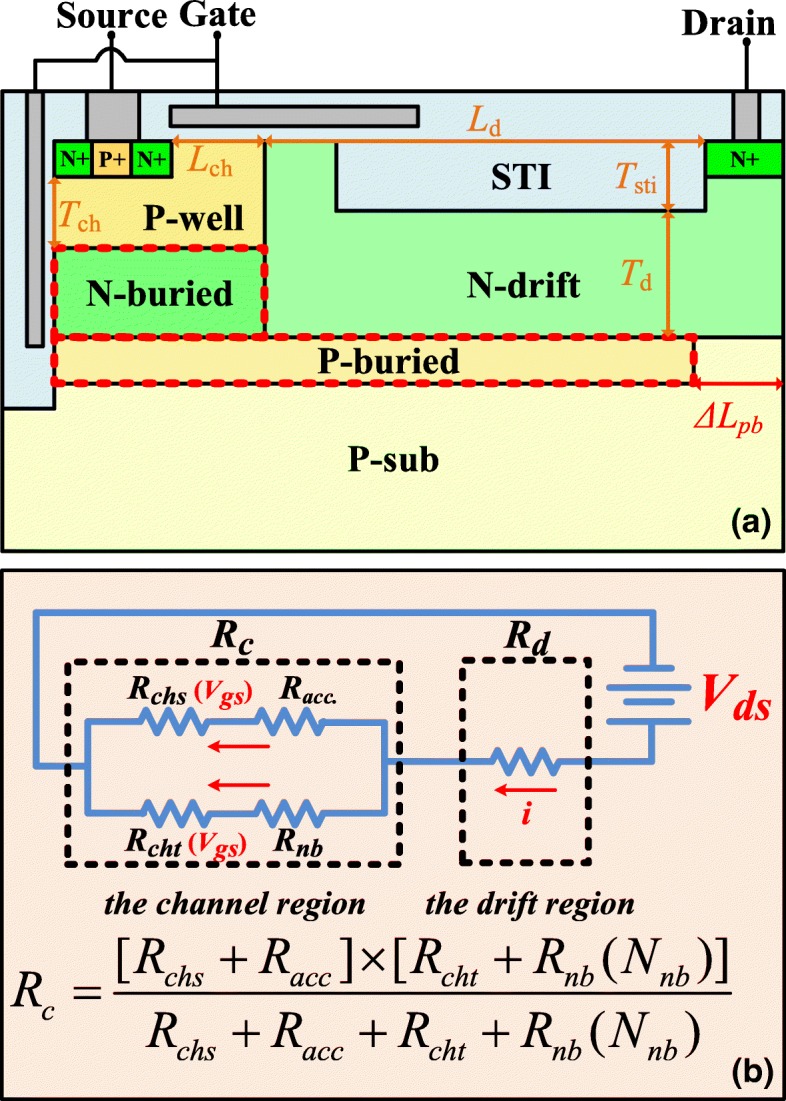
**a** Schematic cross-section view of ultra-low specific on-resistance LDMOS with enhanced dual-gate and partial P-buried layer. **b** Schematic equivalent on-resistance for the proposed LDMOS

Figure 1a shows the schematic cross-section of ultra-low specific on-resistance LDMOS with enhanced dual-gate and partial P-buried layer. The LDMOS features the dual-gate with N-buried layer and the partial P-buried layer which contributes to reduce *R*_on,sp_ and enhance BV, respectively. In the channel region, the enhanced dual-gate is formed by trench gate and highly doped N-buried layer. Compare to conventional dual-gate structure, N-buried layer significantly reduce the resistance of the channel region by provide a low on-resistance conduction path under P-well in the on-state. In the drift region, the partial P-buried layer with high doping concentration is introduced under the N-drift region to enhance BV while maintaining low *R*_on,sp_. The partial P-buried layer helps to reduce the vertical electric field in the off-state without breaking charge balance in the drift region. The key size of the novel device is listed in Table 1.

**Table 1:** The key size of the novel device

*L*_d_	1.6 μm
*L*_ch_	0.3 μm
*T*_ch_	0.2 μm
*T*_sti_	0.3 μm
*T*_d_	0.7 μm

Figure 1b shows the schematic equivalent on-resistance model for the proposed LDMOS. The total on-resistance is considered as the resistance of the drift region (*R*_d_) and the resistance of the channel region (*R*_c_) in series. In the channel region, surface channel conduction path parallels the trench channel conduction path. Thus, *R*_c_ is equal to (*R*_chs_ + *R*_acc_)//(*R*_cht_ + *R*_nb_), where *R*_chs_, *R*_acc_, *R*_cht_, and *R*_nb_ are the resistances of the surface-gate channel, the accumulation region, the trench gate channel, and the N-buried layer, respectively. Based on the proposed on-resistance model, the reduction of *R*_c_ would achieve by decreasing *R*_nb_ without affecting the other performances, because the other resistances are mainly determinate by the process technology, operation voltage, and threshold voltage. The *R*_d_ has been reduced by introducing P-buried layer under N-drift region to enhance the Reduce Surface-field (RESURF) effect in our previous work. In this work, the partial P-buried layer is adopted to improve the BV while maintaining the low *R*_d_.

**Fig. 2:**
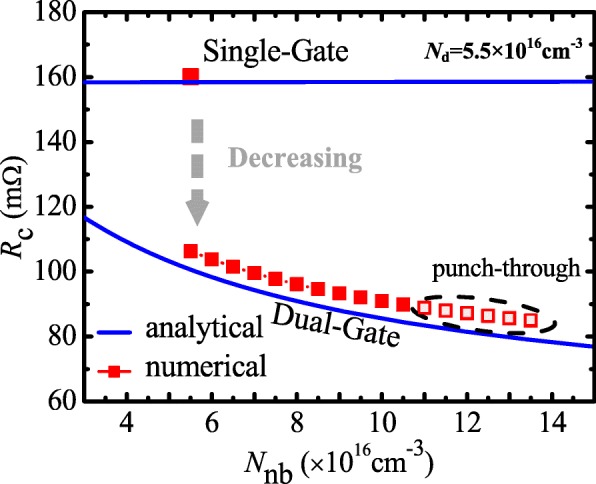
Numerical and analytical *R*_c_ as a function of *N*_nb_ with single-gate and dual-gate (*Z* = 1 cm). *N*_d_ is the doping concentration of the N-drift region

Aiming at the reduction of *R*_c_, the N-buried layer with high doping concentration is introduced under P-well. Figure 2 shows numerical and analytical *R*_c_ as functions of the doping concentration of the N-buried layer (*N*_nb_) with single-gate and dual-gate. It is indicated that the dual-gate structure helps to reduce *R*_c_ compared with the single-gate. When *N*_nb_ = *N*_d_ = 5.5 × 10^16^ cm^−3^, *R*_c_ is 110 mΩ. According to the on-resistance model, *R*_nb_ is the main contributor to *R*_c_. And then, the *R*_nb_ is desired to decrease with the aim of smaller *R*_c_. As shown in Fig. 2a, *R*_c_ is reduced with *N*_nb_ increasing. When *N*_nb_ = 1.35 × 10^17^ cm^−3^, *R*_c_ is reduced to 85 mΩ. However, Fig. 2 also shows that *N*_nb_ would be limited by punch-through breakdown. Because of adding trench gate, *R*_c_ is decreased firstly by 34% with *N*_nb_ = *N*_d_ = 5.5 × 10^16^ cm^−3^. As *N*_nb_ increases, *R*_c_ continuously decreases. With optimized *N*_nb_ = 1.05 × 10^17^ cm^−3^, *R*_c_ is decreased by 45% at last. When *N*_nb_ > 1.05 × 10^17^ cm^−3^, punch-through breakdown will happen in P-well. The analytical result of *R*_on,sp_ shown in Fig. 2 indicates that the proposed model provides a good fitting with numerical simulation results. Therefore, the model is believable to guide the optimization design.

**Fig. 3:**
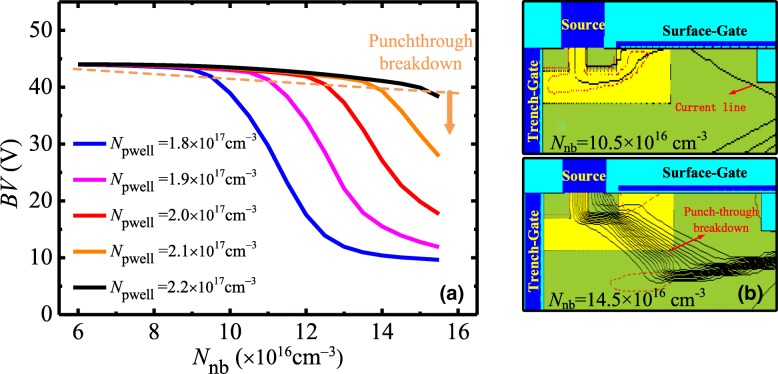
**a** Numerical BV as a function of *N*_nb_ with different *N*_pwell_. **b** Current density profile for *N*_nb_ = 10.5 × 10^16^ cm^−3^ and 14.5 × 10^16^ cm^−3^ while *N*_pwell_ = 2 × 10^17^ cm^−3^ at breakdown

Figure 3a shows numerical BV as a function of *N*_nb_ with different doping concentration of P-well (*N*_pwell_). *N*_nb_ has an effect on not only the *R*_c_, but also the BV. For a given *N*_pwell_, BV keeps unchanged at small *N*_nb_, and then decreases with *N*_nb_ increasing. When *N*_nb_ increases to 1.2 × 10^17^ cm^−3^, BV starts to drop with *N*_pwell_ = 2 × 10^17^ cm^−3^. The drop of BV is ascribed to punch-through breakdown in the P-well region as shown in Fig. 3b. As drain voltage increases, the depletion region in P-well extends to the source. When the depletion region attacks the N+/P-well junction, the punch-through breakdown occurs. For a large *N*_pwell_, the depletion mainly extends to the drift region, and the punch-through breakdown is avoided without degrading the BV. Although P-well with high doping concentration benefits to avoid the punch-through breakdown, it would enhance the threshold voltage. Thus, *N*_pwell_ of 2 × 10^17^ cm^−3^ is chosen with consideration to threshold voltage and the trade-off between the BV and *R*_on,sp_.

**Fig. 4:**
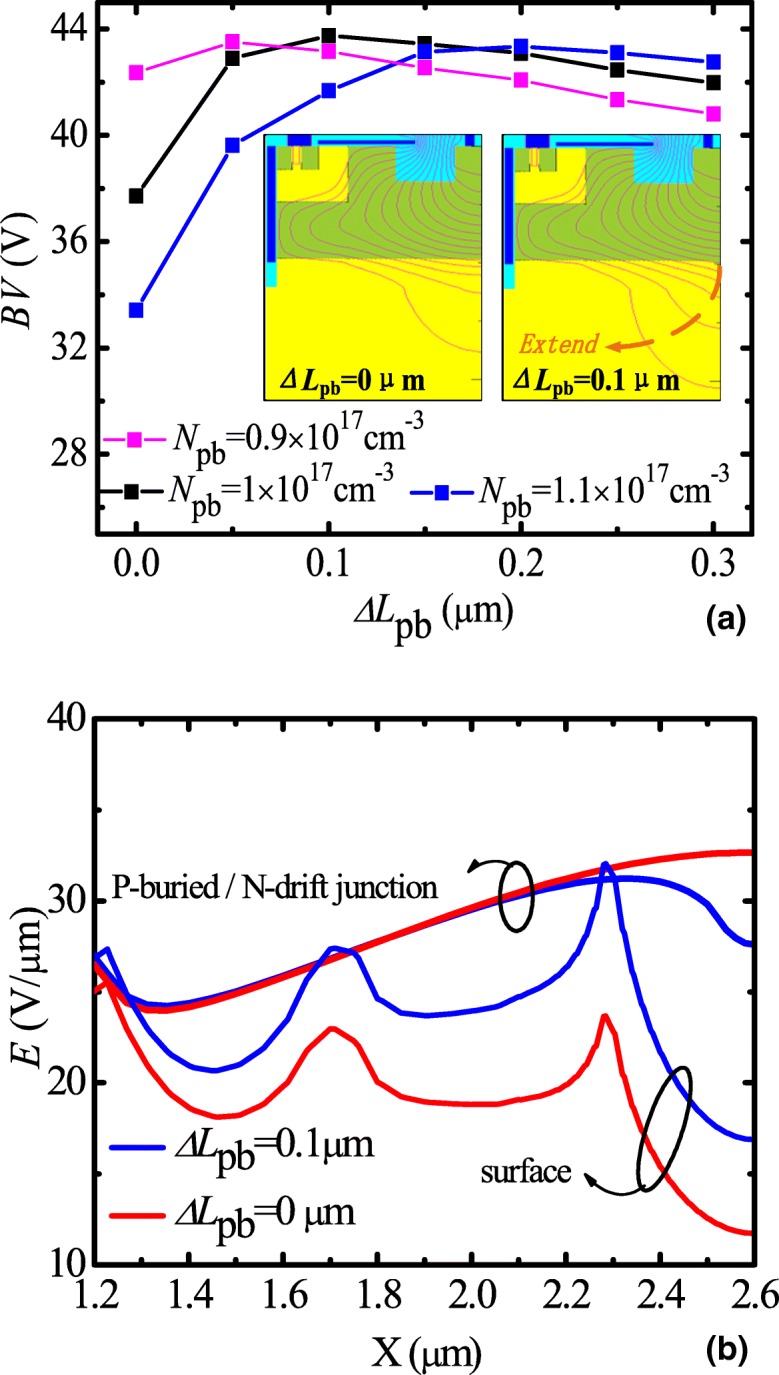
**a** BV as a function of *ΔL*_pb_ with different *N*_pb_. The insert is the equipotential contour profile with *N*_pb_ = 1 × 10^17^ cm^−3^. **b** Electric field distribution at the surface and the P-buried/N-drift junction interface

In order to achieve low *R*_d_ and high BV, partial P-buried layer is introduced under the N-drift region. Figure 4a shows BV as a function of Δ*L*_pb_ with different *N*_pb_. For a given *N*_pb_, as Δ*L*_pb_ increases, BV increases and then decreases slightly. When Δ*L*_pb_ = 0.1 μm, *N*_pb_ = 1 × 10^17^ cm^−3^, BV reaches the maximum value 43 V. The insert shows the equipotential contour profile with *N*_pb_ = 1 × 10^17^ cm^−3^. It is indicated that the equipotential contour in the partial P-buried layer structure extends more to substrate with comparison to full P-buried layer. Figure 4b shows electric field distribution at the surface and the P-buried/N-drift junction interface. For optimized conventional LDMOS, the breakdown occurs usually at the N-drift/P-buried interface. For the proposed LDMOS, the junction of N-drift/P-sub replaces the junction of N-drift/P-buried to relax the vertical electric field and extend depletion region, which results in a higher BV while maintaining low *R*_d_.

**Fig. 5:**
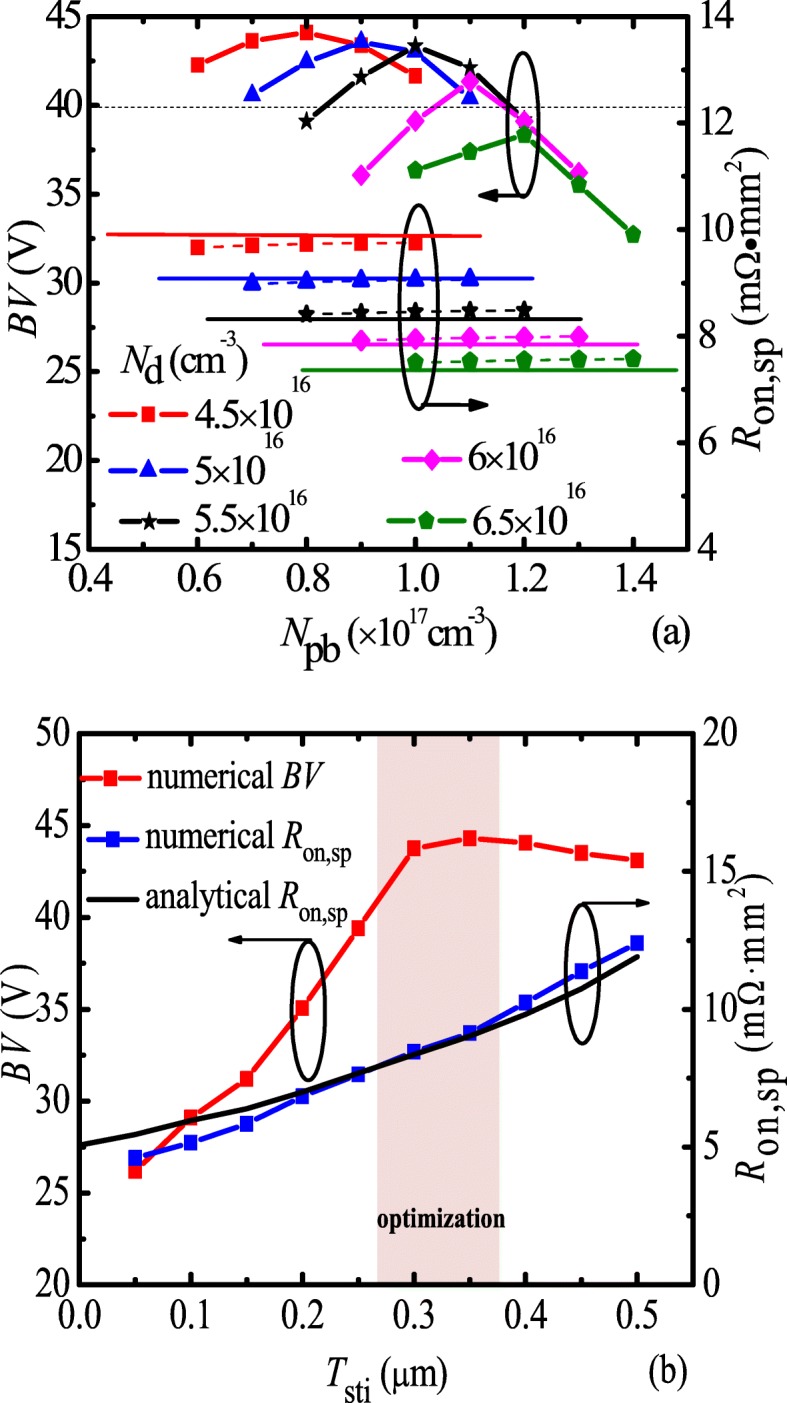
**a** Numerical (dotted line) and analytical (solid line) BV and *R*_on,sp_ as functions of *N*_pb_ for different *N*_d_. **b** Numerical (dotted line) and analytical (solid line) BV and *R*_on,sp_ as functions of *T*_sti_

Charge balance between N-drift and partial P-buried layer is required to achieve high BV. Figure 5a shows that numerical and analytical BV and *R*_on,sp_ as functions of the doping concentration of the P-buried (*N*_pb_) for different *N*_d_. For a given *N*_d_, BV has a maximum value with varied *N*_pb_, and the maximum of *BV* increases with the decrease of *N*_d_. However, *R*_on,sp_ can be increased as the *N*_d_ decreasing. Due to *BV* required higher than 40 V, the *N*_d_ = 5.5 × 10^16^ cm^−3^ and *N*_pb_ = 1 × 10^17^ cm^−3^ are chosen. Figure 5b shows numerical and analytical BV and *R*_on,sp_ as functions of the thickness of the STI layer (*T*_sti_). *T*_sti_ has strong impact on BV and *R*_on,sp_, and it should be designed and optimized carefully as well as our previous work [21]. For *T*_sti_ < 0.3 μm, the breakdown point under the edge of poly field plate has a high electric field peak. As *T*_sti_ increases, the electric field peak is relaxed, and then *BV* increases. For *T*_sti_ = 0.3 μm, BV of 43 V is obtained. For *T*_sti_ ≥ 0.3 μm, the electric field peak under the edge of poly field plate is enough low, as a result, the breakdown point transfers to P/N junction under the drain side. As *T*_sti_ increases, BV increases and then saturates.

**Fig. 6:**
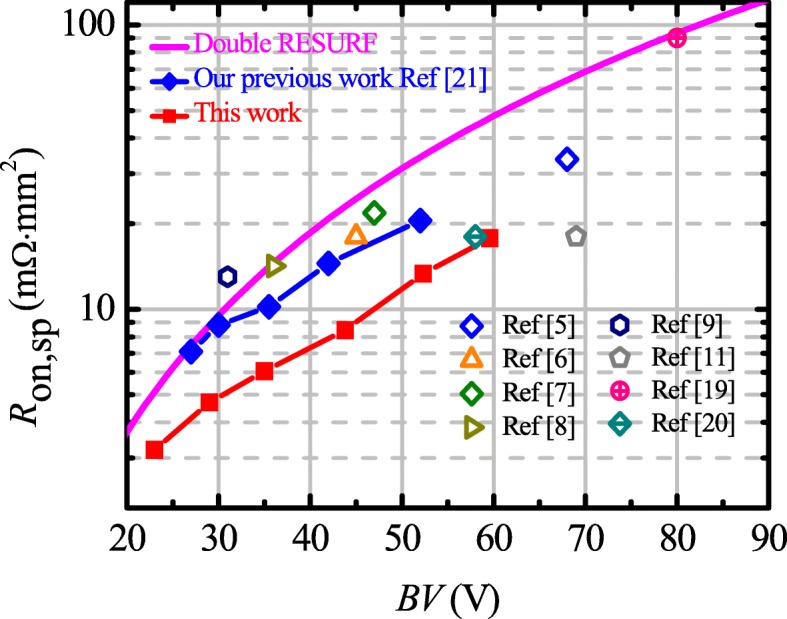
The benchmark of existing BCD technologies and the proposed LDMOS

Figure 6 shows the benchmark of existing Bipolar-CMOS-DMOS (BCD) technologies and the proposed LDMOS. Apparently, the process technology for proposed LDMOS is compatible with our developed BCD technology which achieved the best-in-class performance of LDMOS. In the fabrication process for the proposed LDMOS, N-buried layer could share the same mask with P-well. For the proposed LDMOS, *R*_on,sp_ is 8.5 mΩ·mm^2^ while BV = 43 V, which is reduced by about 37% compared with our previous work.

## Conclusion

A novel ultra-low specific on-resistance LDMOS with enhanced dual-gate and partial P-buried layer is proposed and investigated by numerical simulation in this paper. N-buried layer with high doping concentration is utilized to achieve enhanced dual-gate with reducing *R*_c_. Partial P-buried layer is introduced under the N-drift region to enhance *BV* with keeping charge balance. The fabrication process of the LDMOS in this work is compatible with the existing BCD technology reported in our previous work. The result shows that the *R*_on,sp_ of the proposed LDMOS is reduced by 37% at BV of 43 V compared with previous work. With the semiconductor processing technology going to nanometer level, the *R*_on,sp_ can reduce further with channel length decrease.

## Abbreviations

BCD: Bipolar-CMOS-DMOS
BV: Breakdown voltage
LDMOS: Lateral double-diffused metal-oxide-semiconductor transistor
RESURF: Reduce surface-field
*R*_on,sp_: Specific on-resistance
USTI: Ultra-shallow trench isolation
